# A Comparative Performance Analysis of Computational Intelligence Techniques to Solve the Asymmetric Travelling Salesman Problem

**DOI:** 10.1155/2021/6625438

**Published:** 2021-04-17

**Authors:** Julius Beneoluchi Odili, A. Noraziah, M. Zarina

**Affiliations:** ^1^Department of Mathematical Sciences, Anchor University Lagos, Lagos, Nigeria; ^2^Faculty of Computing, Universiti Malaysia Pahang, Pekan 26600, Malaysia; ^3^Centre for Software Development & Integrated Computing, University Malaysia Pahang, Pekan 26600, Pahang, Malaysia; ^4^Faculty of Informatics and Computing, Universiti Sultan Zainal Abidin, Kuala Terengganu, Malaysia

## Abstract

This paper presents a comparative performance analysis of some metaheuristics such as the African Buffalo Optimization algorithm (ABO), Improved Extremal Optimization (IEO), Model-Induced Max-Min Ant Colony Optimization (MIMM-ACO), Max-Min Ant System (MMAS), Cooperative Genetic Ant System (CGAS), and the heuristic, Randomized Insertion Algorithm (RAI) to solve the asymmetric Travelling Salesman Problem (ATSP). Quite unlike the symmetric Travelling Salesman Problem, there is a paucity of research studies on the asymmetric counterpart. This is quite disturbing because most real-life applications are actually asymmetric in nature. These six algorithms were chosen for their performance comparison because they have posted some of the best results in literature and they employ different search schemes in attempting solutions to the ATSP. The comparative algorithms in this study employ different techniques in their search for solutions to ATSP: the African Buffalo Optimization employs the modified Karp–Steele mechanism, Model-Induced Max-Min Ant Colony Optimization (MIMM-ACO) employs the path construction with patching technique, Cooperative Genetic Ant System uses natural selection and ordering; Randomized Insertion Algorithm uses the random insertion approach, and the Improved Extremal Optimization uses the grid search strategy. After a number of experiments on the popular but difficult 15 out of the 19 ATSP instances in TSPLIB, the results show that the African Buffalo Optimization algorithm slightly outperformed the other algorithms in obtaining the optimal results and at a much faster speed.

## 1. Introduction

There have been disagreements among computer science experts with regards to what constitutes artificial intelligence and computational intelligence [1]. Meanwhile, some researchers argue that artificial intelligence and computational intelligence are one and the same branch of knowledge, and other experts feel that computational science is a branch of the artificial intelligence [2, 3]. Still another school of thought believes that artificial intelligence is a branch of computational intelligence [4]. For the purpose of this paper, the authors are in agreement with the school of thought such as the IEEE Computational Intelligence Society that believes that computational intelligence is a branch of artificial intelligence that focusses on the smartness of a computer machine in terms of performing functions usually attributed to human beings only [5]. Some of those functions include the exhibition of learning, reasoning, making of informed choices, and self-improvement. These are achieved through the development of algorithmic techniques that deploys intelligent agents drawn from the simulation of nature to solve real-life problems. Computational intelligence methods have been applied to solve classification problems, time-series prediction problems, examination scheduling [6], stock market problems, weather forecasting, national economic forecasting, job scheduling [7], electric voltage regulation, agricultural production, industrial site location, vehicle-routing, etc. [8, 9].

On the contrary, artificial intelligence refers to that computer software which enables robots or computer systems perform human tasks with exceptional abilities, accuracy, speed, and capacity. AI encompasses the design of algorithmic and nonalgorithmic methods that involve the use of robots, computer vision, graphics and human-computer interactions, language processing, etc. [10, 11]. Major application areas of AI include the development of novel ways of interactions between man and machines such as the design of better-functioning business models, medical and bio-medical applications, and engineering designs [3, 12].

### 1.1. Need for Computational Intelligence Methods

In view of the enormous contributions of AI and computational intelligence (CI) to human development over the years as highlighted above, researchers have devoted much resources and time investigating this area with a viewing to unraveling the untapped potentials inherent in AI and CI, respectively [13]. Resulting from these research efforts is the realization that AI and CI rely a lot on the effectiveness and efficiency of the methods. This understanding has led to the development of CI methods. In the last three decades, the concentration of researchers on developing swarm and evolutionary optimization methods towards enhancing industrial, scientific, and engineering applications is worth investigating [14]. The constant need for faster, cheaper, and more efficient ways of solving industrial, commercial, engineering, and logistics problems has made optimization a favoured area of scientific investigations in the past few decades leading to the development of novel computational intelligence methods [15].

In recent times, several heuristics cum metaheuristic methods have been designed to solve the problem of optimization in science, engineering, industrial, and technological problems encountered in many practical fields of human endeavour. Some of these optimization techniques are deterministic while others are stochastic. Deterministic techniques/algorithms have in-built mechanisms that guarantee the exact solution to an optimization problem but many times run into serious problems as the search space gets larger [16]. Deterministic algorithms are quite inefficient in multimodal search environments [17]. Some of the deterministic optimization methods are the Finite Difference [18], Hooke–Jeeves pattern [19], Nelder–Mead simplex [20], and Newton–Raphson method [21] to mention but a few. Similarly, among the stochastic optimization techniques are the African Buffalo Optimization (ABO) [22], Max-Min Ant System (MMAS) [23], Model-Induced Max-Min Ant Colony Optimization (MIMM-ACO) [24], Cooperative Genetic Ant System (CGAS) [25], Randomized Insertion Algorithm (RAI) [26], Improved Extremal Optimization (IEO) [27], etc.

Stochastic algorithms use search agent or agents in their search and obtain solutions iteratively without a guarantee of optimal results. However, stochastic algorithms are highly efficient in monomodal and multimodal search environments of any size. Presently, much attention is focused on the stochastic algorithms and, most recently, in hybridization of stochastic algorithms since they tend to be more successful in finding optimal or near-optimal solutions to some difficult real-life situations that require optimization for better results [28]. In this paper, our interest is in comparing the efficiency of different stochastic optimization methods to solving the Asymmetric Travelling Salesman Problems (ATSP).

### 1.2. Hybrid, Metaheuristic, and Heuristic Algorithms

Hybrid algorithms are simply a combination of two or more algorithms in such a way that the algorithms are made to cooperate and jointly solve a problem. Hybridization of algorithms are done to harness the unique capabilities of the cooperating algorithms to enhance search efficiency and effectiveness in terms of obtaining optimal or near-optimal solutions, escaping stagnation and ensuring faster computational speed, etc. There are several algorithm-hybridization architectures in literature ranging from master-slave, relay to peer-to-peer paradigms, etc. In all, algorithm-hybridization synergizes algorithms in such a way as to complement one another in order to ensure greater efficiency and effectiveness [29].

On the one hand, metaheuristics refer to a kind of high level, stochastic, problem-independent, and intelligent manipulators of heuristic information to achieve greater efficiency in their search enterprise [16, 30]. To achieve this, sometimes, metaheuristics accept worsening moves, generate new starting solutions for the embedded local search component or introduce some kinds of memory or experiential biases to ensure the quick production of higher quality solutions, etc. [31]. Examples of metaheuristics are the Ant Colony Optimization [32], Artificial Bee Colony [33], Particle Swarm Optimization [34], African Buffalo Optimization [35], etc.

A heuristic, on the other hand, is an approximate, problem-dependent set of instructions, methods, or principles designed to solve a problem at a reasonable computational cost. Generally, heuristics are, relatively, simple mechanisms designed to determine the cheapest/best/most effective solution among a set of solutions. However, due to the prevalent use of greedy search strategy, heuristics have the problem of premature stagnation. Examples of heuristic algorithms are Lagrangian Relaxation [36], Randomized Insertion Algorithm [37], Greedy Search [38, 39], etc.

The primary difference between heuristics and metaheuristics is that, usually, heuristics are problem-dependent, but the metaheuristics are general-purpose algorithms. Again, metaheuristics have inherent memory capabilities that enable them learn while executing, thus enabling them adapt to any problem, unlike pure heuristic algorithms [40].

To the best of our knowledge, this is the first time the algorithms used in this comparative analysis are being compared together in one study. The analysis in this paper involves hybrid, metaheuristic, and heuristic algorithms. Moreover, this study aims at adding to the body of knowledge in the ATSP literature which, we observed earlier, is not as researched into as its symmetric counterpart though ATSP has more real-life applications than the symmetric TSP. Moreover, it is hoped that it will be a useful tool in the hands of researchers having to carry out studies that involve the ATSP.

The rest of this paper is organized as follows.  Section 2 discusses the Travelling Salesman Problem.  Section 3 introduces the six comparative algorithms, namely, the African Buffalo Optimization (ABO), Cooperative Genetic Ant System (CGAS), Model-Induced Max-Min Ant Colony Optimization (MIMM-ACO), Max-Min Ant System (MMAS), the Improved Extremal Optimization (IEO), and the Randomized Insertion Algorithm (RAI), highlighting each algorithm's basic flow and search mechanisms for the ATSP.  Section 4 concentrates on the experiments performed and discussion of the results obtained in comparing the first five algorithms which are population-based algorithms.  Section 5 compares the performance of the ABO with the RAI. This is followed by the conclusions, acknowledgment of support for the study, and references.

## 2. Travelling Salesman Problem

The travelling Salesman Problem (TSP) is about the most studied problem among combinatorial optimization problems and is fast becoming the most reliable test bed for newly designed optimization methods [41]. The Travelling Salesman Problem which was designed, developed, and popularised by Eric Weisstein at Wolfram Research around 1930 [11], basically, is the problem of a particular salesman who has customers all over a large city requiring his services or products. To satisfy these customers, the salesman has to visit all of them and then return to the starting location using the cheapest route. The TSP, in a way, is comparable to a graph theory problem where the arcs represent the routes/roads and the nodes represent the cities. TSP, therefore, is a Hamiltonian cycle where the cycle passes through all the nodes, at least, once in the graph [42]. The arcs in a TSP problem are assigned some weights which represent the costs/distances/travelling time between the nodes in order to help in determining the arc that has the cheapest cost [37].

There are two types of TSP: asymmetric and symmetric. Usually, the symmetric TSP is easier to solve since both to and from journeys are the same in cost/length, as such optimization algorithms simply calculate one length of the journey across different nodes [43]. In the asymmetric TSP, there exists an instance, at least, where the cost/weight on an arc is not the same in either to or from a node in the travelling graph [44]. Meanwhile, in the symmetric TSP, the travelling cost/time/weight is the same on either way in all the graphs [45]. That is to say that, in the asymmetric TSP, the edges in the to and from direction can have different costs/time periods/weights/lengths. As such the problem should be modeled with the aid of a directed graph. In the symmetric, however, the distance between any pair of nodes is the same in either direction. TSP can be represented mathematically as TSP={(*G*, *f*, *t*), where *G*=(*V*, *E*), *f* is a function *V* × *V*⟶*Z*, and *t* ∈ *Z*. As such, *G* is a complete graph that fully represents the tour of the travelling salesman with the entire tour cost which should not exceed *t*.

In ATSP, there exists a set *V* of cities coupled with a cost function *c* that represents the weights between any pair of nodes, *V* × *V*⟶*R*+, with a requirement to find a minimum tour length/cost that ensures that every node is visited at least once. The constraint of visiting a city at least once as opposed to exactly once is relevant because, usually, the starting city is visited twice. Thus, the ATSP tour can be represented as

(1) $$\begin{array}{c} c\left(T\right)=\sum_{a\in T}^{n}\left(c\left(a\right)\right). \end{array}$$

This way, the ATSP tour of any three cities *u*, *v*, *w* ∈ *V* satisfies the triangular inequality. This means that the following statement holds for the ATSP tour:

(2) $$\begin{array}{c} c\left(\left(u,w\right)\right)\leq c\left(\left(u,v\right)\right)+c\left(\left(v,w\right)\right). \end{array}$$

Available literature indicates that metaheuristic approaches used in solving the TSP with a little transformation are effective in providing solutions to the ATSP [46, 47]. The same cannot be said of heuristics that are mostly problem-dependent. Similarly, many researchers assert that the ATSP is more difficult to solve than its symmetric counterpart because it requires a reformulation as a symmetric TSP problem with some constraints [48, 49].

### 2.1. The Need for Asymmetric TSP

A critical review of literature on the Travelling Salesman Problem reveals that there are lots of studies on the symmetric Travelling Salesman Problem over the past several decades. However, it is rather ridiculous that there exists a paucity of literature on the asymmetric TSP [50]. This is rather puzzling because most day-to-day human activities are, indeed, asymmetric. Consider, for instance, a postmaster delivering mails to different locations within a large city or even to different geographical zones, a school bus driver picking up school children and returning the children at the end of school period, a taxi driver picking up passengers from the taxi station and returning for his next queue, and welfare officers taking food to home-bound persons [51]. In all of these cases, the most probable route would be asymmetric. This study is motivated by the indispensable nature of asymmetric challenges in virtually all aspects of human endeavour. It is hoped that the study will find wide practical applicability. Asymmetry in logistics and transportation in real-life settings could result from one-way traffic situations and road tolling, as well as other commercial and/or civil engineering considerations [51].

## 3. The Comparative Algorithms

This study specifically investigates the performance of six optimization algorithms in literature that have exhibited exceptional performances in solving the ATSP. These algorithms are the African Buffalo Optimization (ABO), Model-Induced Max-Min Ant Colony Optimization (MIMM-ACO), Max-Min Ant System (MMAS), Cooperative Genetic Ant System (CGAS), Improved Extremal Optimization (IEO), and Randomized Insertion Algorithm (RAI). The choice of these algorithms for comparison is informed by their special characteristics, while the ABO and the IEO are standalone metaheuristic algorithms, the MMAS, MIMM-ACO, and CGAS are hybrid metaheuristics, and the RAI is a heuristic algorithm.

### 3.1. African Buffalo Optimization

**Figure 1:**
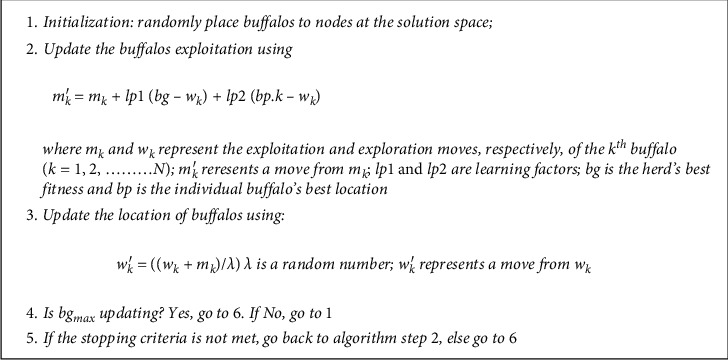
ABO algorithm.

The ABO which was inspired by the marvelous organizational ability of herds of buffalos, which, sometimes, are upto 1000 individuals in a single herd, using two primary vocalizations: the *waaa* and the *maaa* [26, 30], is a relatively new algorithm whose search capacity cum ability to obtain good results is very competitive. The ABO applying the lean metaheuristic design concept was designed to be fast in obtaining results, avoid stagnation, use few parameters, and be efficient and effective; hence, it is the choice for this comparative study. It was actually designed to complement the existing algorithms such as the Genetic Algorithm [52], Simulated Annealing [53], Ant Colony Optimization [54], and Particle Swarm Optimizations [55]. Using these vocalizations, the African buffalos organize themselves in their navigation through the African forests in search of lush green pastures to satisfy their huge appetite [35]. In this algorithm, each animal's location represents a solution in the search space. The ABO algorithm is presented in Figure 1.

The ABO applies the Modified Karp–Steele algorithm in its solution of the Asymmetric Travelling Salesman Problem [56]. It follows a simple solution step of first constructing a cycle factor *F* of the cheapest weight in the *K* graph. Next, it selects a pair of arcs taken from different cycles of the *K* graph and patch in a way that will result in a minimum cost. Patching is simply removing the selected arcs in the two cycle factors and then replacing them with cheaper arcs and, in this way, forming a larger cycle factor, thus reducing the number of cycle factors in graph *K* by one. Thirdly, the second step is repeated until we arrive at a single cycle factor in the entire graph *K* [57, 58].

So far, the observed limitations of the ABO lie in the fact that buffalos are parliamentary in decision-making. That is to say that the choice of majority population of the herd determines their next destination. In the standard ABO variant, the modeling process is not explicit leading to the generation of a new population when there is a case of stagnation occasioned by the decision of the majority of the herd. Another area of weakness is that the frequent reinitialization of the entire population has the tendency to limit the directional search capacity of the buffalos when the algorithm is faced with complex engineering challenges. These observed challenges necessitated the development of the Improved African Buffalo Optimization [59].

### 3.2. Cooperative Genetic Ant System

The Cooperative Genetic Ant System (CGAS) [25] is a hybrid algorithm that combines the Genteic Algorithm (GA) with the Ant System (AS) in a concurrent and cooperative manner, thus harnessing the individual strengths of both algorithms in ensuring search efficiency. In solving the ATSP, CGAS selects the next node for an ant to visit based on natural ordering and selection. Resident in any node is a sorted list of a certain number adjoining nodes that are chosen through natural selection process in such a way that a node with higher probability is chosen for the next move. For any ant to move from one node to another, the ant will consult the sorted list *C*(*i*) to pick the nearest node in a process represented by

(3) $$\begin{array}{c} j=\min c\left(i\right),\mathrm{if}\;j\in Sk,\arg\max\tau ij\eta ij,\beta ,\text{otherwise}. \end{array}$$

**Figure 2:**
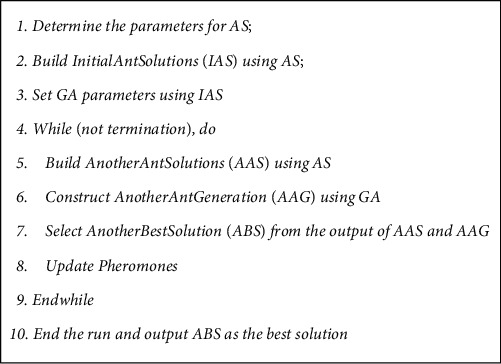
CGAS algorithm.

This information exchange between GA and AS in the end of the current iteration enables the algorithm to choose the best solutions for the next iteration. Such cooperation helps the algorithm to arrive at the global optimal solution and ensures adequate exploration of the search space. The Cooperative Genetic Ant System algorithm is presented in Figure 2.

### 3.3. Max-Min Ant System

**Figure 3:**
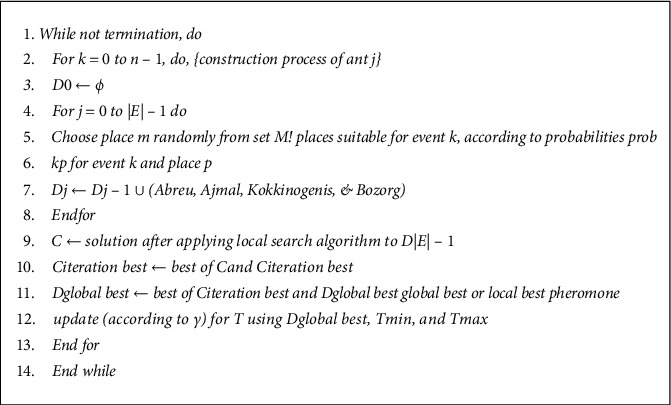
MMAS algorithm.

The Max-Min Ant System (MMAS) developed by Stuzzle and Hoos [60] is simply an extension of the classical Ant Colony System (ACS) algorithm by ensuring that only the best ant in each iteration or the global best ant (i.e., the ant with the best solution since the beginning of the search) is allowed to deposit pheromone along its own route. At the start of the algorithm, the pheromone trail is set to some maximum levels to ensure adequate exploration, but this is systematically reduced as the algorithm progresses. This system is akin to the Ant System but is further extended by placing a limit to the quantity of pheromones depositable to a particular maximum and minimum (max, min) values on the chosen arcs/routes. This is to avoid the problem of stagnation observed in the Ant System and the Ant Colony Optimization where so much pheromone is deposited on some favourite arcs/route, thereby, diverting the attention of the ants from exploring other parts of the search space. The maximum and minimum pheromone trail values are selected in a problem-dependent manner, such that the more promising routes are given, the higher max-min values are obtained. By the end of each iteration, the evaporation factor reduces the strength of the pheromone trail by a given factor but making sure that the trail used by the best ant is given less evaluation. This ensures that the pheromone trail strength on less-promising arcs decreases, thus directing the ants to more promising arcs. The MMAS algorithm is presented in Figure 3.

To solve ATSP, first, the ants are placed on some randomly selected nodes/vertices and they start constructing their tours from an initial node deliberately exploiting the pheromone trails *rkj* associated with each edge (*k*, *j*). Such a tour is constructed by choosing the next vertice probabilistically using

(4) $$\begin{array}{c} P_{kj}∼T_{kj}^{\alpha },\mathrm{if}\;j\text{ is not yet visited},\text{otherwise,}\text{0}. \end{array}$$

### 3.4. Improved Extremal Optimization

**Figure 4:**
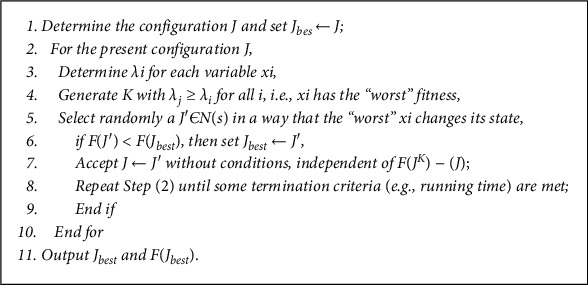
Improved extremal optimization algorithm.

The Extremal Optimization algorithm [61] was inspired by the self-organised critically (SOC) system theory which is a combination of two models that use different extremal dynamics: the Bak–Sneppen (BS) model and BTW sand-pile model [62]. The Extremal Optimization (EO) is closely associated with the BS evolution model which is akin to natural biological evolution that favours species with higher fitness values. The EO algorithm differs from the other evolutionary algorithms in which it emphasizes cooperative co-evolution and extremal dynamics in the evolutionary process. In solving the ATSP, the nodes/cities are mapped into a multientity physical system in such a way that, for an ATSP problem with *k* cities, there exists *k* − 1 different states. The entire anticipated solution that includes all nodes is deemed a state in the physical system. Next, the algorithm defines a local fitness function that evaluates the energy of each entity in the physical system. In every iteration, the algorithm moves through two major phases, namely, the extremal dynamics and the cooperative co-evolution phases which are combinations of greedy search and random walk. The EO algorithm is presented in Figure 4:

There has been a major modification of the classical Extremal Optimization algorithm: the Improved Extremal Optimization [27] which introduces the parameter *τ*that is adjustable, meaning that EO algorithm Step (2) is slightly changed in a way that the variable with the *n*th highest fitness is chosen with the probability *Pj* ∝ *j* − *τ* (1 ≤ *j* ≤ *N*) especially in a situation when there are *N* entities in the computational system.

### 3.5. Model-Induced Max-Min Ant Colony Optimization

Model-Induced Max-Min Ant Colony Optimization (MIMM-ACO) [63] is a hybridization of the Max-Min Ant System algorithm with Karp's Patching technique [64] and the Patch heuristics [65] resulting in two main adjustments to the classical Max-Min Ant System algorithm (MMAS). First, the static pheromone weighting system is replaced by a dynamic pheromone weighting mechanism. The dynamic weighting mechanism results from the partial path construction efforts of the ants. In this way, deliberate attempt is made to favour more promising edges (i.e., edges that have lower residual costs rather than just mere lower actual cost, thus eliminating the nonoptimal edges) of the search. Secondly, MIMM-ACO algorithm termination condition rather than being determined intuitively is determined analytically based on the present structure of the pheromone matrice in a particular iteration, thus enhancing the algorithm's searching capacity.

**Figure 5:**
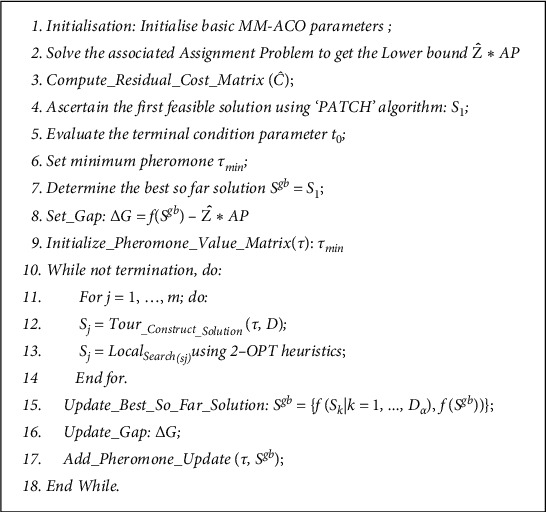
MIMM-ACO pseudocode.

In solving the ATSP, the algorithm, first, analyzes the ATSP problem and then applies the MIMM-ACO searching system. Information obtained from this phase is then used to direct the search further towards more promising areas of the search space. The pseudocode code of MIMM-ACO is presented in Figure 5:

From Figure 5, *D* is the cost matrix, *Ĉ* is the residual cost matrix, *s*^gb^ is the best solution obtained so far, and *τ* represents the pheromone matrix.

### 3.6. The Randomized Insertion Algorithm

**Figure 6:**
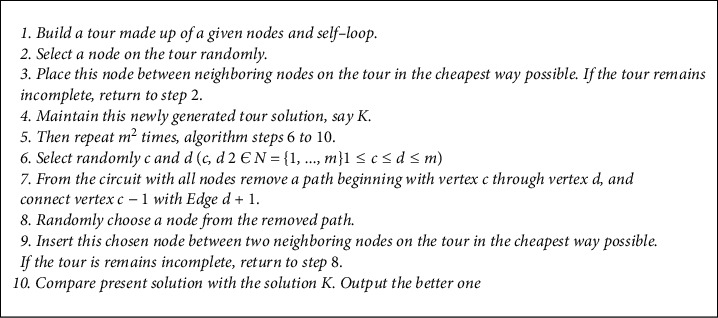
RAI algorithms.

The randomized algorithms make random instead of deterministic decisions through the extensive use of random bits as their input in its search process, thus leading to the generation of random variables. Randomized algorithms are usually faster and simpler than deterministic ones. The Randomized Insertion Algorithm (RAI) uses the arbitrary insertion mechanism which is very close to cheapest insertion strategy in its search for solution to the ATSP. The development of the RAI was borne out of a desire to provide a fast and simple solution to the ATSP. This algorithm starts by constructing an initial solution (see steps 1–4 in Figure 6) and then employing a series of systematic deletion cum insertion of arcs in the cheapest way possible as it constructs good solutions.

To solve the ATSP, the RAI randomly selects any initial node *a* and then links it with any two other nodes *c* and *d* in the cheapest possible way thereby forming cycle *ac*  *d*. In the next iteration, the RAI selects any other cheap node/nodes within the neighborhood of the newly formed cycle which is not part of the already-formed tour and inserts such into the tour randomly. This process is repeated until all the nodes are inserted. Next, the algorithm keeps this tour and proceeds to the deletion phase (see steps 6–10 in Figure 6) where the algorithm randomly deletes some arcs while comparing the present solution with the previous, retaining the better, and discarding the worse. At the end of the construction steps, the algorithm calculates and outputs the best solution found.

## 4. Experiments and Discussion of Results

The experiments were performed using a desktop with the following configuration: Intel Duo Core ™ 2.00 Ghz, 2.00 Ghz, 1 GB RAM on a Window7 on 15 difficult but popular instances out of the 19 Asymetric Travelling Salesman Problem (ATSP) dataset ranging from 17 to 443 cities available in TSPLIB95 [66]. The experiments were coded in Matlab programming language and executed on Matlab2012b compiler.

### 4.1. Parameter Setting

The details of the experimental parameters are available in Table 1. The explanation of the symbols used in Table 1 is as follows. *D*^*∗*^ is the dimension of the problem, that is, the number of nodes; *α* represents the pheromone factor; Ǫ is the pheromone amount; *β* is the heuristic factor; *D*^*∗*^ is the size of population; *qo* is the exploitation ratio; *ρ* is the pheromone evaporation parameter; *ϕ* is the ratio of minimum to maximum pheromone value; *w*min is the minimum value of biased weight; *θ* is the termination condition parameter; N/A denotes Not Available; *τ* is the probability selection; *N*_ITER_ is the maximum number of iterations. Please recall that *m*_*k*_ and *w*_*k*_ represent the exploitation and exploration moves, respectively, of the *k*th buffalo (*k*=1,2,…*N*); *m*_*k*_′ represents a move from *m*_*k*_; lp1 and lp2 are learning factors; bg is the herd's best fitness; bp is the individual buffalo's best location. The parameters are set after deliberate tuning. For the ABO specifically, since it is a parameterless algorithm, the parameters are preset by the algorithm designers [57].

The parameters were obtained after careful parameter-tuning. The parameters used in this experiment are found to give the best results. Please note that, to ensure fairness of comparison among different algorithms, it is necessary to run the experiments in the same machine and using the same programming language.

### 4.2. Computational Results

The comparative experiments were of two parts: the first compared the output of the metaheuristic algorithms in solving the ATSP, while the second compared the ABO performance with that of the RAI which is a heuristic algorithm. The results of the experiments involving the metaheuristic algorithms, namely, the Model-Induced Max-Min Ant Colony Optimization (MIMM-ACO), Max-Min Ant System (MMAS), Improved Extremal Optimization (IEO), Cooperative Genetic Ant System (CGAS), and African Buffalo Optimization algorithm (ABO) are presented in Table 2.

Please note that the relative error was obtained by

(5) $$\begin{array}{c} \text{Rel}.\text{ error}=\frac{\text{Best}-\text{opt values}}{\text{Opt values}}\times 100. \end{array}$$

In Table 2, the best performer is the MIMM-ACO. The algorithm obtained optimal result in all the 15 ATSP cases under consideration here. This excellent performance was closely followed by the IEO, CGAS, ABO, and MMAS in that order. As pointed out earlier, all these algorithms posted excellent results in solving the ATSP. In fact, to the best of our knowledge, they presently hold some of the best results in the literature in solving the ATSP, and this is the motivation for this comparative study.

On the whole, all the algorithms posted over 94.56% accuracy in solving the problems. These are excellent performances, especially when one realises that these are metaheuristic algorithms, that is to say, they are general-purpose algorithms that were not specifically designed for just the ATSP. One way to explain their exceptional performances could be that they are all hybrid algorithms, except, of course, the ABO. Hybrid algorithms post good performances since they exploit the strength of individual algorithms being hybridized. ABO's good result could be traceable to its use of less complicated calculation of fitness function coupled with the ability of the buffalos to search both globally and locally at the same time.

In terms of the computational cost which is judged by the amount of computational resources utilized in obtaining the solutions to the ATSP instances under investigation, this is where there is such a gulf in the algorithms performances. Here, the exceptional performer is the ABO. It took the ABO just 20.58 seconds to solve all the ATSP instances under investigation. The next best performer is the MIMM-ACO with 78.51 seconds. These are commanding performances, especially when we consider that it took the other algorithms hundreds of seconds to solve the same number of problems. The excellent performance of the ABO could be due to its use of relatively few parameters. Basically, the algorithm uses two major parameters, the “*waaa*” and “*maaa*” vocalizations of the buffalos, to control its flow. MIMM-ACO good solutions could be traceable to the introduction of the limit parameters of the MMAS and the inherent search ability of the classical ACO in addition to the excellent constructive ability of the patching technique (see Table 2).

It could be observed from Table 2 that the other algorithms were, rather, very slow. Solving the same problems took IEO 392.03 seconds, MMAS 492.39 seconds, and CGAS 780.22 seconds. Overall, the ABO is over 3.8 times faster than the MIMM-ACO, 19.05 times faster than IEO, 23.93 times faster than MMAS, and over 37.91 times faster than CGAS. The slow speed of these algorithms could be due to common problems with algorithm hybridization. In most cases of hybridization, efficient exploration is either sacrificed for greater exploitation, speed for optimal solution, or vice versa. Moreover, due to the complicated hybrid algorithm architecture, more parameters that require tuning and more complex implementational skill requirement from hybridization, and hybridization poses a threat to efficiency [67]. Since efficiency (speed) and trustworthiness (accuracy) are two of the major hallmarks of a good algorithm, the others being versatility and ease of use [68], it is safe to conclude that the ABO having obtained over 98.5% of the optimal results of all the ATSP instances under investigation and being, clearly, the fastest of all the algorithms under consideration, and it may be safe to conclude that the ABO is a better algorithm.

## 5. African Buffalo Optimization and Randomized Insertion Algorithm

The previous analysis of the performance of the metaheuristic algorithms shows that the ABO has an edge over the other metaheuristics. This section is concerned with the comparative assessment of the performance of the ABO with the RAI heuristics in solving the ATSP. The RAI heuristics was especially designed to provide solutions to asymmetric TSP instances. The experimental results are presented in Table 3:

The two algorithms, under investigation in Table 3, the ABO and the RAI, posted very commanding performances. While the ABO obtained over 98.6% accuracy in all 15 ATSP instances, the RAI obtained over 99.05% accuracy. Moreover, it can be observed that the ABO obtained the optimal solution in five instances to RAI's 13 accurate performance. The difference in performance here can be traceable to their use of two different techniques in obtaining results. While the RAI uses the random insertion strategy, ABO uses the modified Karp–Steele method. Nonetheless, it is a competitive performance.

The excellent performance of both algorithms is further highlighted by the calculation of their cumulative relative errors which is a measure of deviation from the optimal solutions. The cumulative relative error is obtained by summing up the values of the relative errors for each ATSP instance. The cumulative relative error of the ABO is 1.32% and that of RAI is 0.94%. This is also a commendable performance by the ABO in view of the fact that the RAI is a pure heuristic designed primarily to solve the ATSP.

In evaluating the cost implications of obtaining results, the uncommon strength of the ABO becomes outstanding in all instances. It was only in Br17 that the RAI executed slightly faster in 0.027 seconds to ABO's 0.028 seconds. In the remaining 14 instances, the ABO clearly outperformed the RAI. For instance, while it took ABO 0.037 seconds to obtain result in Ry48p, the RAI used 1.598 seconds. This means that the ABO was over 43.18% faster. This trend continues throughout the remaining ATSP instances under investigation. In fact, the ABO gets progressively faster as the number of ATSP cities increases. Take, for instance, the two largest city instances here which are Rbg403 and Rbg443, while ABO used 4.741 and 10.377 seconds, respectively, the RAI used 11137 and 17126 seconds, respectively. This shows the ABO being over 2,349 and 1,650 times faster, respectively.

As was the case in the comparative performance of the metaheuristics, it can be seen that the ABO outperformed the heuristic algorithm, RAI. Someone may have observed that speed is a function of the hardware configuration, the programmers' expertise, and a few other factors; nevertheless, an algorithm that has such a straightforward calculation of fitness function and uses very few parameters will undoubtedly obtain results faster than most other algorithms. In all, aside from ABO's capacity to obtain over 98.5% accuracy to RAI's 99.06%, it took ABO a total of 20.582 seconds to to RAI's 38979.448 seconds to execute all the 15 instances under investigation.

## 6. Conclusion

This study examined the solutions to the asymmetric Travelling Salesman Problems using computational intelligence techniques. The computational intelligence techniques used include African Buffalo Optimization algorithm (ABO), Improved Extremal Optimization (IEO), Model-Induced Max-Min Ant Colony Optimization (MIMM-ACO), Max-Min Ant System (MMAS), and Cooperative Genetic Ant System (CGAS), as well as the heuristic and Randomized Insertion Algorithm. Experimental results obtained from using these algorithms to solve the ATSP reveal that the MIMM-ACO performed excellently obtaining the optimal solutions to all test instances. However, it was discovered that, to obtain such an excellent result, the MIMM-ACO sacrificed speed. It took the MIMM-ACO 78.51 seconds to solve the 15 ATSP instance, while another algorithm, the African Buffalo Optimization (ABO), obtained 98.6% accuracy at 20.582 seconds. The study, therefore, concludes that since efficiency (speed), trustworthiness (accuracy), versatility, and ease of use are hallmarks of a good computational intelligence methods [68] and a number of experimental evaluations with focus on the first two criteria, the ABO is adjudged a better algorithm for solving the ATSP instances, followed by MIMM-ACO.

The excellent performance of the MIMM-ACO is traceable to two main factors. First, the algorithm's ability to replace static biased costs/weights in an ATSP with dynamic ones is something other algorithms struggle to do. This ability stems from the algorithm's use of partial solutions sampling that each ant has constructed in course of the search and then discarding less fruitful results while holding on to the very best. Moreover, the MIMM-ACO's use of the assignment problem technique in discarding the nonoptimal solutions from the list of available solutions is a major advantage. Second, MIMM-ACO determines the final output based on the most recent state of the pheromone matrix and combines this using the patch algorithm to micromanage the solutions obtained by the assignment problem. Other algorithms find it hard to outperform the MIMM-ACO's hybridization of the assignment problem with the patch. This basically explains why the MIMM-ACO results in ATSP remain one of the best over the years [63].

It is recommended that the other algorithms should be fine-tuned to make them faster. Moreover, the authors recommend the comparison of the performance of ABO with other state-of-the-art algorithms in providing solutions to other optimization problems such as knapsack problem, graph coloring, and urban transport challenges in major cities. Finally, in view of the relevance of the ATSP to our every day activities, it is recommended that more research efforts should be directed towards solving ATSP and its practical applications in transportation, logistics, national security architectural challenges, etc.

### 6.1. Threats to Validity

As much as the algorithms in this comparative study performed excellently well, it must, however, be observed that good results are a function of the programming language used for the study as well as the machine used for the experiments. Moreover, the choice of benchmark test cases could be a threat that the algorithms performed well in these chosen benchmarks may not be a guarantee that they will do well in other benchmarks. Again, the choice of the comparative algorithms could be a threat that these algorithms performed well against one another may not guarantee their exceptional performance when compared with other newer algorithms.

Finally, it is possible that the programming expertise of our programmer as well as the programming language used in implementing this study could have influenced our experimental output.

## Figures and Tables

**Table 1:** Experimental parameter setting.

ABO		MIMM-ACO		IEO		MMAS		CGAS	
Parameter	Value	Parameter	Value	Parameter	Value	Parameter	Value	Parameter	Value
Population	40	Ants (*n*)	10	Population	*D* ^*∗*^	Population	*D* ^*∗*^	Generation	100
*λ*	2.0	*β*	2.0	*N*_ITER_	200000	*β*	5.0	*β*	2.0
lp1	0.6	*ρ*	0.1	*τ*	3.0	*ρ*	0.99	*ρ*	0.1
lp2	0.5	*α*	1.0	*α*	Cost	*α*	1.0	Ro	0.33
N/A	1.0	Ǫ	200	*B*	Best	Φ_*ij*_	rand (−1, 1)	Crossover rate	1.0
N/A	N/A	N/A	N/A	N/A	Known cost	N/A			
—	N/A	*Qo*	0.85	N/A	N/A	*Qo*	0.9	*qo*	0.9
N/A	N/A	Φ	1/*n*	N/A	N/A	*Ǫ*	200	*ϕr*	0.3
N/A	N/A	*w*min	1.001*ϕ*	N/A	N/A	N/A	N/A	*ϕρ*	0.2
N/A	N/A	*θ*	1.5	N/A	N/A	N/A	N/A	*τ*_min_	*τ*_max_/20
N/A	N/A	N/A	N/A	N/A	N/A	N/A	N/A	*τ*_max_	1 − (1 − *ρ*)
Total no of runs	50	—	50	—	50	—	50	—	50

**Table 2:** Comparative experimental results of metaheuristics on ATSP.

MIMM-ACO			MMAS		IEO		CGAS		ABO	
TSP case	Rel. err %	CPU time (s)	Rel. err %	CPU time (s)	Rel. err %	CPU time (s)	Rel. err %	CPU time (s)	Rel. err %	CPU time (s)
Br17	0	0.01	0	0.0	0	0.01	0	0.01	0	0.028
ft53	0	3.53	0.22	3	0	3.85	0.35	6.78	0	0.028
ftv33	0	6.12	0	10	0	4.78	0	28.73	0.08	0.029
ftv35	0	5.35	0	15	0	7.35	0	21.35	0.07	0.030
ftv38	0	8.64	0	11	0	7.83	0	29.79	0	0.026
ftv44	0	9.37	0	12	0	8.21	0	37.63	0.06	0.032
ftv47	0	7.52	0	10	0	9.37	0	29.7	0.06	0.029
ftv55	0	6.38	0	19	0	5.06	0	18.41	0.12	0.029
ftv64	0	15.37	0	28	0	16.42	0	29.25	0.0	0.041
p43	0	8.35	0.08	9	0.13	5.47	0	7.53	0.44	0.065
ry48p	0	7.83	0	8	0	5.45	0	12.35	0.12	0.037
rgb323	0	0.01	1.3	97	0.06	87.12	0.13	103.28	0	2.050
rgb358	0	0.01	0.75	75	0	69.65	0.35	96.49	0.18	3.043
rgb403	0	0.01	1.35	104.39	0	85.32	0.31	147.83	0.08	4.741
rgb443	0	0.01	1.73	91	0	76.14	0	143.76	0.11	10.37
Mean	0	15.5	0.36	32.83	0.013	26.14	0.08	52.02	0.09	1.37
Total	**0**	**78.51**	**5.43**	**492.39**	**0.19**	**392.03**	**1.24**	**780.22**	**1.4**	**20.58**

Rel. err = relative error; CPU time = total time taken by the algorithm to obtain result; s = seconds.

**Table 3:** Comparative experimental results.

ATSP cases	No of cities	Opt	ABO				RAI			
			Best	Avg	Rel. er %	Time	Best	Avg	Rel. er %	Time
Br17	17	39	39	39.98	0	0.028	39	39	0	0.027
Ry48p	48	14422	14440	14455	0.12	0.037	14422	14543.20	0	1.598
Ftv33	34	1286	1287	1288.4	0.08	0.029	1286	1288.16	0	0.393
Ftv35	36	1473	1474	1475.8	0.07	0.030	1473	1484.48	0	0.508
Ftv38	39	1530	1530	1536.4	0	0.026	1530	1543.12	0	0.674
Ftv44	45	1613	1614	1647.25	0.06	0.032	1613	1643.6	0	1.198
Ftv47	48	1776	1777	1783	0.06	0.029	1776	1782	0	1.536
Ft53	53	6905	6905	6920.25	0	0.028	6905	6951	0	2.398
Ftv55	56	1608	1610	1618.2	0.12	0.029	1608	1628.74	0	2.878
Ftv64	65	1839	1839	1938	0	0.041	1839	1861	0	5.241
P43	43	5620	5645	5698	0.44	0.065	5620	5620.65	0	0.997
Rbg323	323	1326	1326	1417.75	0	2.050	1335	1348	0.68	3874
Rbg358	358	1163	1187	1299.2	0.18	3.040	1166	1170.85	0.26	6825
Rbg403	403	2465	2467	2475	0.08	4.741	2465	2466	0	11137
Rbg443	443	2720	2723	2724	0.11	10.377	2720	2720	0	17126
Total	—	—	—	—	**1.32**	**20.582**	—	—	**0.94**	**38979.448**

Opt = optimal values as recorded in TSPLIB; Best = best results obtained by a particular algorithm; Avg = average values obtained after 50 runs; Rel. er (%) = relative error percentage; Time = time taken by the CPU to obtain results.

## Data Availability

The data used to support the findings of the study are available within the article.
